# Mental health, subjectivity and the city: an ethnography of migrant stress in Shanghai

**DOI:** 10.1093/inthealth/ihz029

**Published:** 2019-05-30

**Authors:** Lisa Richaud, Ash Amin

**Affiliations:** 1 Health Communication Institute, School of Public Health, Fudan University, PO Box 248, 138 Yixueyuan Road, Shanghai, Shanghai; 2 Department of Geography, University of Cambridge, Downing Place, Cambridge, UK

**Keywords:** Ethnography, Everyday stress, Mental health, Subjectivity, Urban China, Urbanicity

## Abstract

Ethnography, with its focus on everyday experience, can yield significant insights into understanding migrant mental health in contexts where signs of severe mental distress remain largely imperceptible, and more generally, into how stresses and strains are lived through the spaces, times and affective atmospheres of the city. Migrant ethnography can help us reconsider the oft-made connection between everyday stress and mental ill health. In this contribution, drawing on field evidence in central and peripheral Shanghai, we highlight the importance of attending to the forms of spatial and temporal agency through which migrants actively manage the ways in which the city affects their subjectivity. These everyday subjective practices serve to problematize the very concept of ‘mental health’. The paper engages in a critical dialogue with sociological and epidemiological research that assesses migrant mental health states through the lens of the vulnerability or resilience of this social group, often reducing citiness to a series of environmental ‘stressors’. Distinct from methods ascertaining or arguing against the prevalence of mental disorders among urban migrants, the insight of urban ethnography is to open up a space to explore the mediations that operate dialogically between the city as lived by migrants through particular places and situations and forms of distress.

## Introduction

This article seeks to intervene in ongoing debates about urban living, stress and mental health^1–3^ with specific attention to precarious contexts.^4^ It highlights the significance of an approach that attempts to get close to the actual lived experience through which mental states are formed, as subjects encounter and engage with their urban environment in situated contexts.^5^ The article asks: How does a close-up account of the ways in which precarious lives are lived complicate common associations between urban environments and mental ill health? Drawing on ethnographic data collected as part of a multi-method project, ‘Migration, Mental Health and the Chinese Mega-City’, it explores the insights of a method that looks beyond prevailing approaches focusing on the likeliness of (rural) migrants to experience mental disorders, mild and severe, in the ‘alien’ city, enabling inductive, ethnographic inquiry into the ways in which migrants experience and inhabit their urban lifeworlds. At the core of this enterprise is an attempt to reconceptualize the ways in which the city and its residents (in our case, Chinese internal migrants in Shanghai) are mutually shaped.

China’s economic reforms (post-1978) and fast-paced urbanization have brought unprecedented movements of rural populations to the cities in search of work and opportunities to ‘see the world’ (*jian shimian*). In Beijing, Guangzhou and other mega-cities, several generations of internal migrants have been engaging in wage labor, petty entrepreneurial activities, or small or more upmarket trade. As such, ‘migrants’ by no means constitute a homogeneous group, as neither do ‘migrant entrepreneurs’, ‘migrant workers’ and other subcategories defined by different positions in the labor market (see Zhang^6^). Yet, because of China’s household registration system (*hukou*) which divides the population into two categories, ‘agricultural’ and ‘urban’, migrants generally share a legal status that ties them to their rural birthplace and restricts their access to education,^7^ welfare,^8^ housing^9^ and entitlement to temporary residency permits in the cities. This disadvantaged positioning, often paired with poor working and housing conditions, has prompted much discussion about how urban life translates into mental health and well-being.^10–13^ In the prevalently epidemiological, psychological and sociological literature, identified ‘risk factors’ or ‘stressors’ generally include ‘social exclusion’, ‘stigma’ or ‘victimization’, as well as ‘economic pressure’, ‘migration stress’ and ‘isolation’, all of which are believed to frame much of migrant experience in the city.^14^ Relying on large-scale surveys, these accounts are often framed in terms of vulnerability and resilience,^15^ resulting in classifications of migrants as ‘mentally unhealthy’ or otherwise (see Li and Rose for a review^16^).

As urban ethnographers writing on the mental life of migrants, we perceive a paradox in much of this literature. While scholars veer towards negatively interpreting the assumed defining characteristics of rural-to-urban everyday migrant experience (e.g. overcrowded and cramped residential environments, harsh and precarious working conditions, social alienation and stigma, and bewilderment at the fast and furious pace of life), rarely do they seek to grasp the actual embodied experiences of research participants beyond the identified set of discrete stressors or factors to which urbanicity is reduced. If this scholarship draws our attention to correlations between symptoms of mental ill health and generic categories of risk, what still goes amiss is a careful understanding of how lived urban environments feel like to those who inhabit and engage with them, and how, in this process of inhabitation, mental states are formed. Yet affective and embodied experience can hardly be read from labels: what is conceptualized as mere stressors may obscure multiple realities and situations to which migrants are not passively exposed, and whose implications for mental life could be far from straightforward or reducible to a positive or negative impact.

These considerations lead to a second and related point. While factors such as ‘social exclusion’, ‘stigma’, ‘migration stress’ and the like have been recurrently identified as hallmarks of migrant experiences of the city across an array of studies, we would caution against the reduction of the urban everyday as lived by migrants to such factors. Without denying the importance of conditions such as socioeconomic disadvantages, institutional barriers, labor rights infractions or the injuries caused by prejudice, it would be an omission to play down the significance of the many ordinary moments that make up migrant lives, which social identities and structural positions do not exhaust. Migrant lives are not only lived through difficult experiences and perceptions relating to institutional and material circumstances, social status and belonging, hopes and expectations, or inner deliberations over the ‘meaning of migration’.^15^ The minutiae of the everyday, we would argue, are not trivial details: disturbing easy equations between urban life, stress and mental health, they may well constitute the very site through which migrants learn to negotiate their precarious conditions, rendering them more habitable.

We came to this view as the realities in the field forced us to move beyond a narrow focus on mental ill health, and because readers may find the ethnographic material presented below at best loosely related to mental health despite our project’s topic being framed in such terms, some clarifications are in order. In its incipient stage, ‘Migration, Mental Health and the Chinese Mega-City’, the larger project in which our research is embedded, had two ambitions. One was to approach the forms of distress potentially experienced by migrants, and largely described in the literature, through thorough investigations of everyday life in the metropolis. The aim was to understand the relationships between the lived city and mental states, or the negotiation thereof. The second ambition was to engage with people experiencing mental illness, living with a diagnosis, or seeking help in healthcare services. Here again, the objective was to understand how the city plays out in the lives of people dealing with mental health issues. Because the dynamics in the field seldom mirror research projects, the initial plan proved difficult to carry out. Not only did the institutional context constrain access to migrant residents (without Shanghai *hukou*) dealing with such issues, but migrants themselves rarely sought help in those healthcare services which consented to provide assistance in our research project. Moreover, when conducting ethnographic observation of migrant lives in the field sites described below, mental distress did not arise as a particularly salient issue among a majority of our interlocutors living and getting by in Shanghai. We did not come across obvious or severe forms of mental distress and disorder beyond everyday stress and anxiety, sleeplessness and ‘thinking too much’^17^ or depressed moods (neither did one member of our research team who conducted a questionnaire survey in both areas). These symptoms often did not constitute major obstacles against participation in social life.

Therefore, what we observed in our field sites (to be introduced below) begged for a return to the kind of basic questions referred to earlier: How does the urban everyday feel for our interlocutors? If life in the metropolis is often associated with ‘stress’ and ‘pressure’ (*yali*) in their discourses, does this condition translate into unpleasant feelings as one works, rests, takes care of family members and interacts with friends and neighbors? What does the flow of the urban everyday do to those carrying worries, anxiety and troubled thoughts on a moment-to-moment basis? Underlying these questions is a reflection on what stress means and does in the urban experience of migrants. In this article, drawing on the work of the psychological anthropologist, Victoria Burbank,^18^ we use ‘stress’ to refer to those unpleasant affects and feeling (some of which may be described as stress in emic terms, although this may not necessarily be the case) potentially arising as a result of specific social, institutional and economic arrangements. Crucially, we understand the emergence of these bodily states as mediated by the specific moment-to-moment situations in which people find themselves. Because situations are necessarily dynamic, so is the experience of stress. There is a need, we believe, to elucidate this, before we go on to equate everyday stress with risks of mental illness, for the forms of negotiation of stress through ordinary situations may well disturb this very equation. This is where we find our ethnographic data relevant to ongoing discussions on urban mental health in the context of China’s internal migration and beyond, echoing recent reviews of epidemiological studies that point to the lack of such in-depth accounts (see Manning^19^; Li and Rose^16^).

In shedding light on possibilities that remain amidst the constraints posed to migrant lives, we have no desire to participate in discourse that normalizes inequalities, but instead to suggest that skills of negotiation ought to be given a more prominent place in inquiry about ‘migrant mental health’. This contribution, therefore, cautions against overlooking how the city is experienced and lived, and asks about that which comes to the fore in attending to the sensorial and the affective tonalities of daily life in observing mundane social activities unfolding in a variety of urban settings. This is something that anthropology, with its reliance on ethnographic methods, is particularly well equipped to explore. However, this is not to suggest that further research of this kind be left to anthropology alone, as it has important implications for the very ways we think about ‘migrant mental health’ and for public health research at large. The broader question of how the city is implicated in the making of inner selves should be of much wider disciplinary and professional interest, for it has important implications for mental health policy and practice in cities.

## Introducing the field

### A place-based approach

Spanning a period of 15 months between January 2017 and October 2018, ethnographic fieldwork in Shanghai was carried out in two contrasting areas of the city. Located in Songjiang District on the south-western outskirts, the first area can be described as a low-rise migrant neighborhood structured around one main shopping street and a market square, where a large majority of the shops and small trades are run by migrants, along with a school for migrant children and one large residential center for the floating populations. The street was surrounded by factories as well as old houses owned by Shanghai-born residents and rented out to migrants. A few months after our fieldwork began, the factories were torn down in large numbers in late 2016 as a result of a local government decision to remove illegal and polluting works in a bid to ‘green’ the local economic base. This sudden and massive transformation generated considerable uncertainty among the largely migrant residents, most of whom were middle-aged men and women who had lived with their families and worked in the area for over a decade, either as petty entrepreneurs (*getihu*) in the informal sector, as low-paid workers, such as street cleaners and security guards, or as manual workers employed in nearby factories. While the bulk of our investigations were carried out between January and July 2017, additional fieldwork was conducted between August and October 2018 in a nearby small-scale electrical motors factory which had survived the urban redevelopment program.

Situated in the middle of the inner-city Huangpu District, in proximity to some of the city’s most famous tourist attractions, such as the Bund or Nanjing Road, the second fieldwork area consists of two large blocks of old low-rise buildings and narrow alleys inserted in between Shanghai’s high-rise commercial district, with its upscale malls and office buildings. Much in contrast to the suburban migrant neighborhood, the aura of cosmopolitanism appealed to our informants, many of whom were born after the 1980s and 1990s, and worked in ‘bijou’ cafés, upmarket stores or restaurants which became our field sites. Despite its ongoing gentrification, the neighborhood remains socially heterogeneous, and the space of its many bookstores and library was regularly appropriated by casual laborers or unemployed men, who also became the focus of our study.

### Participant observation and informal talk

In both areas, we relied on participant observation to approach social life in all its complexity, in the workplace, in public space, or (when possible) within houses and worker dormitories. Central to ethnographic investigations, participant observation entails ‘establishing a place in some natural setting on a relatively long-term basis in order to investigate, experience and represent the social life and social processes that occur in that setting’.^20^ In more concrete terms, the ethnographer gets immersed in the lifeworlds of their interlocutors as they go about their daily activities and routines. While it is common for ethnographers to live alongside the people they work with, the first author of this piece (who conducted most of the empirical work) was living in the northern part of the city and traveled to the field sites several days a week. The degree of involvement as one participates in local life varies according to the role one manages to construct and is ascribed by one’s interlocutors through repeated interactions. Although disclosing one’s identity as a researcher, the ethnographer can be an onlooker in a card game, a fellow participant in a dance gathering, a regular patron in a café, a friend or acquaintance sitting at the family table for dinner or helping with small tasks...When conducting fieldwork in each field site, hundreds of people were observed in a variety of settings, and dozens of them were engaged in conversation, either regularly or occasionally. While the ethnographer usually seeks to establish relations of trust with those alongside whom one works, engagements are of varied intensities. As some anthropologists have noted, this is especially true in urban contexts, where social relations can imply fleeting, light-touch modes of interactions, as well as ‘special-purpose relationships’.^21^ Although the ethnographer has to remain attuned to those various modes of interaction for what they say about specific living contexts, the consequence is a ‘detailed knowledge of some areas of [one’s interlocutors’] lives and almost none of others’ (see Ferguson^21^).

Despite such limitations, participant observation efficiently allows the ethnographer to gain insights into the everyday making of local worlds (i.e. the ‘what’, ‘when’, ‘who’ and ‘how’ of action), as well as to access their informants’ own perceptions of and feelings about their environment and life events. In this respect, informal talk is an integral part of participant observation. Bearing in mind the anthropologist Dell Hymes’s^22^ admonition that ethnographers should not assume that ‘what there is to find out can be found out by asking’, very often in our case, informal verbal exchanges ranging from ‘small talk’^23^ to more intimate conversations were deemed more appropriate than semi-structured interviews, which often proved unsettling to those with whom we had become acquainted or established friendships. Such informal conversations are important not only because they enable the ethnographer to discuss those themes which are relevant to the research projects, but first and foremost because they offer a way to find out, through careful listening, about what matters to the people one works with.

### Field notes

Participant observation entails the writing of field notes, which are central to ethnographic practice.^24^ They record, through 100s of pages, the sayings and doings of one’s interlocutors, as well as the ethnographer’s subjective thoughts and feelings about ongoing events in the field. The production of field notes is most likely to take place ‘behind the scenes’;^20^ however, whenever possible, in-situ note-taking was used, to produce detailed descriptions and ‘sensory imagery’^24^ of ongoing activities such as card games or street encounters. The aim was to document ordinary, yet always singular, situations, through which specific individuals live and by which they are affected. Beyond the focus on individuals per se, these descriptions help to provide a sense of the rhythms and atmospheres of place, of what people make out of their environment.

The field notes were analyzed using the ‘open’ and ‘focused’ coding methods as advocated by Emerson and colleagues.^24^ While in the former, ‘the ethnographer reads fieldnotes line-by-line to identify and formulate any and all ideas, themes, or issues they suggest, no matter how varied and disparate’, the latter consists in ‘subject[ing] fieldnotes to fine-grained, line-by-line analysis on the basis of topics that have been identified as being of particular interest’. In the corpus of field notes produced through observations in Songjiang District, key themes included emptiness and boredom, uncertainty and the importance of remaining optimistic. In the city center, achievement, ‘dreams’ or disillusion were recurrent topics. In what follows, however, we do not present a systematic, exhaustive account of the themes identified through coding. While staying close to our interlocutors’ concerns, we simultaneously highlight aspects and processes which our informants themselves did not necessarily verbalize, but which came to the fore as we reflected retrospectively on migrant lives from the specific perspective of stress, mental health and the city.

## ‘Thresholds of life’, managed stress and the ecologies of endurance

In both areas, life in Shanghai is certainly associated with pressure or stress (*yali*) by migrant residents, who mainly use the term to refer to financial difficulties caused by a general order of things in the metropolis that involves low incomes, high rents and cost of living. *Yali* indexes an external condition one confronts on a daily basis, but that only in moments translates into bodily and mental states. For in the meantime, we found our interlocutors navigating amidst the constraints and possibilities of the city, striving and surviving, anguishing over the present and future, but also living, resting, playing and socializing. To be clear, we do find instances of the usual concerns and injuries often described as typical of migrant lives. But equally important perhaps is how the affordances of the material and social environment leave one dealing with bleakness, by engaging in various social activities, moving between different ‘thresholds of life’, an expression we borrow from the work of the medical anthropologist, Veena Das.^25^ Although Das herself does not offer a definition, we find this expression to aptly convey the idea that individuals do not simply move from one mood or mental state to another. Rather, life as lived involves more subtle shifts, through which one may carry on, bearing ‘the marks of suffering endured’ while finding the energy to engage in ordinary activities.^25^ As Das^25^ writes: ‘Many people within the same environment move from one threshold of life marked by bleakness, even abjection, to some other threshold at which they seem to engage with others, laugh, eat, have sex, look after children, greet visitors’. We understand these ordinary experiences as crucial to the extent to which individuals let themselves be affected by their condition.

Among countless examples of such ‘subtle movements’,^25^ we might cite that of a young mother taking torn clothes to a seamstress working outdoors and recounting how, in the factory where she is employed, the owner has suppressed the possibility for her and her colleagues to work paid extra hours, demanding that productivity be increased during regular hours. This is certainly an expression of stress, a corporeal consequence of *yali* experienced in a specific situation, paired with frustration and anger. But in the course of the interaction, the ‘visceral complaint’^26^ becomes modulated into something else, as she adjusts to the affective tonality of a casual chat and jokes with a familiar one.

Beyond the suburban migrant neighborhood where the spatial ordering and rhythms of daily life facilitate such interactions with acquaintances and friends, we find many other examples of stress (here again, understood as bodily felt states) absorbed into the ordinary in the seeming anonymity of the city center, although under different forms. Every day, casual workers and unemployed men, some of whom are disabled, loiter in one room of the public library; a gathering that clearly is not a group. Where a quick look could easily lead to resorting to the figure of the ‘isolated’ migrant to make sense of the situation, closer observation reveals forms of sociality, in which what counts is not the pleasure of verbal interaction but the very sharing of silence and calmness. Such forms of public solitude should not be mistaken for isolation; it is a mode of being-with, a quiet negotiation of existential difficulties. In the library, for instance, one middle-aged, divorced man from Zhejiang Province, who occasionally engaged in casual labor, had found a place to rest and to maintain normal routines after a period during which the dangers he faced in the aftermath of engaging in illicit activities in his previous life had led him to consider suicide as a potential way out.

If space and time are directly implicated in the movements between different thresholds of life, this is often as the result of some form of spatial and temporal agency, by which we refer to a capacity to act upon, or to do with, space and time. As shown in the case of migrants finding a place for themselves in the library, our informants actively invested bearable contours in their urban environments, acting upon their emotions and mental states in this very process: a corner of the sidewalk in the neighborhood used by amateurs of line dancing and karaoke; a rudimentary room at the residential center for migrants decorated and turned into a gathering place for participants in the weekly meetings of an underground church, where some factory workers indulge in singing sessions or find some relief from adverse family circumstances in a space where tears can be shed silently, although legitimately.

Strikingly, these thresholds of life seem to be incompressible, even under extremely disruptive circumstances. When massive destructions occurred in the suburban area, leaving petty entrepreneurs with both worries and boredom on their hands due to a significant drop in business activity, we found them engaging in various social activities, preserving routines or crafting through casual chats, jokes exchanged or card games. These activities provide temporary relief from overwhelming uncertainty and empty time, certainly not always capable of making boredom and low-key moods fully escapable, but nevertheless endowing the present with ‘meaningfulness’ according to our interlocutors. In rather different cases of employment loss in the inner city, a lonely young man and former café worker negotiates his emotional turmoil and self-doubts, caused by being dismissed from jobs, by finding a place to rest and read amid the shelves of ‘success studies’ books in a bookstore. Or a group of fellow workers, stunned by the sudden closing of the café where they were employed, cheer themselves momentarily by indulging in the pleasures of consumption and flânerie afforded by the inner city, posting pictures of these moments of enforced idleness on WeChat (a popular social media app).

The ways in which encounters with locals (*bendiren*) are involved in the making of these thresholds of life are also worth noting, balancing between prejudice and forms of social recognition. A 27-year-old café manager in the city center feels the reluctance of older Shanghai-born tenants as he looks for a place to rent near his workplace. But the performance of his role at the café allows for other types of affective encounters with locals, as when, for instance, he is offered mitten crabs (a delicacy in Chinese cuisine) by a middle-aged woman whose daily visits to the café result in playful verbal exchanges and unembarrassed laughter amidst the stress of the working day.

Common to many life trajectories we came across in the field, therefore, was a sense of endurance, crafted out of the ecologies through which migrants experience urban life. Certainly, some environments afford a wider range of pleasurable effects than others. But even for workers in the fast-paced service industry, or for those in the motor factory, where the noise of industrial equipment and the ‘squalor’ (to use a term that recurred in the workers’ comments) challenge the senses of the newcomer, there is room for adjustment. Habit (*xiguan*) is invoked as a key process through which endurance is forged, as one learns to disattend or dis-sense, as well as to undertake small acts of self-preservation, finding ways to erect boundaries, albeit often porous, between one’s body and polluting matters. And, in between moments of fatigue, there are the shared meals with fellow workers, the outings to a nearby shopping area, the evening dance gatherings, the cigarette breaks and all that can offer temporary reprieve.

Endurance can take different forms. Those who choose to remain despite the demolitions of their surroundings may occasionally acknowledge their low-key moods, but bad feelings and distress are simultaneously downplayed. For, as many interlocutors explained, dwelling upon one’s suffering is of little help. Our observations suggest that one persists by drawing on every possibility that one’s social environment has to offer to feel otherwise, as evidenced by a 50-year-old seamstress, a mother of two, in the suburban area, an affective response that can endure for years (for more detailed examples, see Richaud and Amin).^27^ Or there is the practice of job-hopping (*tiaocao*) performed by younger migrants in the city center, who carry the exhaustion and frustration accumulated in one workplace to a new one. An eventual return to the home province can offer some relief, when feelings of ‘groundlessness’ (*zhan bu zhu jiao*) and frustration become overwhelming, as was the case for the aforementioned dismissed café waiter who experienced his hiring as a restaurant worker as a form of downward mobility and illustration of the ‘meaninglessness’ of his present life. But this resignation is often only temporary: further plans are made, one goes back to the city and new difficulties are endured. Even when the dream that brought people to Shanghai in the first place is realized by middle-aged workers when they are finally able to build a house in the home province, they continue to stay in the city, perhaps for the additional income they can generate, often for an indeterminate number of years. A potential return is considered, talked about, yet often delayed (see also Zhan).^28^ Pictures of a cozy house in the home province are often displayed with pride on one’s mobile phone, and the contrast with one’s dormitory or tiny room in Shanghai could hardly be more obvious. Yet our interlocutors, who aspired to return home one day, spoke of the ‘needs not to dwell in too good conditions’ in the city. The circumstances are borne with fortitude. This is not to deny complaints about the ‘messiness’ (*yi ta hutu*) of living in a tiny dwelling space in the city, as observed with a woman in her early 50s fumbling among plastic bags and all kind of objects stored on a steel-framed bunk bed in search of something she needed as she prepared dinner. But the poor quality housing that many social surveys mention as sources of mental ill health is endured, sometimes made pleasurable through convivial sociality, as during gatherings or dinners with neighbors and coworkers.

## Studying urban mental health: foregrounding the self-environment nexus

Placed against the dominant tradition of research that emphasizes the suffering, predicament and ‘social exclusion’ of migrants in the city, how does our ethnography of everyday life and stress complicate existing depictions? Looking at factory and service workers, as well as petty entrepreneurs living under heightened uncertainty, we found a certain open-endedness and complexity in terms of mental states formed through urban living. For example, in a scholarly context where migrant mental health is assessed in terms of succumbing to the stresses and strains posed by the city (or sometimes as a form of resilience understood as a competence in the face of risk),^15^ where does one situate the aforementioned middle-aged seamstress, who on her own occasional avowal, ‘can’t really say that she is doing well’ (*shuo hao ye bu hao*) and yet carries on, facing ‘one day after the other’? What of the middle-aged man from Zhejiang Province who whiles away most of his days in a public library in Shanghai’s city center? Many of our cases raise similar questions. Perhaps, as Veena Das has argued in her work on the lives of the poor and the diseased in popular neighborhoods of Delhi,^25^ rather than ‘ask what makes the resilience of some people as opposed to others’, it may be more important to attend to the ‘subtle movements between…different thresholds of life’, some ‘marked by bleakness’ and others imbued with a gentleness of the ordinary. This is the kind of orientation our field findings have demanded, with fluctuations of subjectivity (formed in the intersections of accumulated biography, imagined experience and lived urban materiality) escaping easy labels such as ‘resilient’ or its opposite. Here, subjectivity must be approached as the sum of ‘inner life processes and affective states’,^29^ shaped through engagements with specific environments that return as the sites through which people act on their lifeworld. To frame our inquiries in terms of ‘subjectivity’ rather than ‘mental health’ more suitably acknowledges the open-endedness of affective and psychological states arising out of the constant interplay between escalations of exhaustion, anxiety or stress, and slowing-down processes, mediated by the ways in which the city is inhabited and in turn inhabits the subjects. It thus becomes crucial to attend to how stress and unpleasant feelings are absorbed into the ordinary by migrants, tacitly and reflectively, before jumping to posit causal relationships between urban environments and mental ill health.

What, then, does an emphasis on subjectivity tell us about the co-constitutive relationships between the city and the minds and bodies of its precarious inhabitants? First, by considering migrants as active subjects, we must move beyond conceptions of the city as a pre-given, constraining environment that impacts on individuals, towards understanding it as a series of affective environments that are constantly encountered situationally, through practices of inhabitation and place-making, amidst a varying degree of socioeconomic, material or institutional constraints. Second, an inductive approach questions ‘stressfulness’ as an inherent quality of the city described in much literature. Undeniably, urban dynamics do generate circumstances in which unpleasant feelings are experienced, as previous examples showed. But equally, in many of our sites and situations of urban negotiation, the city as lived through embodied encounters plays an important role in the shifts between the thresholds of life referred to in the previous section of this article. As such, the city itself affords the variation of inner states through which precarious existences are made habitable. Interestingly, sociological and epidemiological surveys have sought to test moderating factors of mental distress, yet rarely interpret them as resources within life as lived through the textures of the urban social space. For example, Lei^14^ identifies the moderating effects of ‘educational attainment’ against the predicaments posed by adverse conditions, while Wong and He^15^ emphasize the ‘meaning of migration’ (i.e. the reasons for living in the city). While, in this latter case, some emphasis is put on the subjectivity of migrants, the interpretive labor in which migrants are said to engage seems to be imagined as an attribute within the confines of the individual, an aspect of the private inner self. In contrast, in proposing subjectivity as always embedded within the environment, and in a constant co-constitutive relationship with it, the sense of endurance that we have identified through our investigations appears crafted out through daily engagements with the city. It is this self-environment nexus that merits further attention in research on migrant lives and mental health, opening a path beyond studies of coping, a term which, as Bister and colleagues^30^ note, has overly individualistic and cognitive connotations.

Perhaps the skeptical reader will be quick to note that we arrive at our conclusion because of the absence of cases of severe forms of mental distress in our sample, perhaps also noting that such distress is often suppressed. Studies have shown, for example, that rural migrants seldom seek clinical help when experiencing psychological distress.^31^ This, along with the absence of the language of ‘mental (ill) health’ among our informants, implies that there may well be hidden evidence of mental distress, suggesting the need for interdisciplinary studies of the kind that Das and and the members of her research team^25^ have undertaken in poor neighborhoods of Delhi, with households carefully surveyed by psychologists and ethnographers. We believe, however, that this limitation does not undermine the significance of the forms of engagement in routines and practices of endurance highlighted here, along with the valuation on the part of our informants of optimism and ‘happiness’ (see Richaud and Amin^27^). Nor does it undermine the subject–environment reciprocity that we have sought to highlight, always posing moods and psychological states as experiential, more than set by the deeper accumulations of life experience and capability. Furthermore, while we make no claims about the statistical representativeness of the ethnographic data, we do think that the situations observed in the field can be the object of what Didier Fassin calls a ‘comprehensive generalization’,^32^ being representative of processes which may occur elsewhere, in China and beyond.

## Conclusion

It is quite possible that the practices of endurance we have emphasized may in themselves constitute a threat, in the longer term, to migrant mental health, in so far as they suppress symptoms that require clinical attention. It is also possible that these practices are always fragile, temporary and generally reflexive. This is very much the gist of what we found in the field: endurance without guarantees. But more broadly, what this research reveals is that the daily experiences of migrants in Shanghai and in other Chinese cities are worth understanding in studies of urban mental health, for they show the limitations of the language of ‘stressors’ such as ‘social exclusion’, ‘migration stress’ or ‘poor quality housing’. They show that the mental states of migrants cannot be read from intimations of social disadvantage or urban habitat. In making a case for an anthropological approach to migrant mental health in the city, our study does not conceive of ethnography as an alternative way to answer the kind of questions upon which epidemiological and sociological works are built. Rather, it shows how the issue can be framed in a different light, on the basis of alternative conceptualizations of migrants in the city.

## Notes

**Authors’ contributions:** LR carried out the main part of the ethnographic fieldwork, except for a 2 wk joint field study with AA; LR and AA undertook the analysis and interpretation of the ethnographic data. LR drafted the manuscript, which was later edited by AA. Both authors read and approved the final manuscript. LR and AA are guarantors of the paper.

**Acknowledgements:** We would like to thank our research team members, as well as our interlocutors in the field.

**Funding:** Economic and Social Research Council Research Grant ES/N010892/1 (Newton fund) and Natural Science Foundation of China Research Grant 71561137001.

**Ethical approval:** Social Sciences, Humanities and Law Research Ethics Subcommittee, King’s College London, reference number: HR-16/17-2887. Verbal consent was obtained from interlocutors in the field and documented in the field notes. Verbal consent was used because some of the research participants were either unable or unwilling to provide this on paper for cultural reasons. Written consent was abandoned as it was severely changing the dynamic of the relationships with the participants.
